# Identifying high-need patients with multimorbidity from their illness perceptions and personal resources to manage their health and care: a longitudinal study

**DOI:** 10.1186/s12875-020-01148-3

**Published:** 2020-04-29

**Authors:** Mieke Rijken, José Maria Valderas, Marianne Heins, Francois Schellevis, Joke Korevaar

**Affiliations:** 1 Nivel (Netherlands Institute for Health Services Research), PO Box 1568, 3500 BN Utrecht, The Netherlands; 2 Department of Health and Social Management, University of Eastern Finland, Kuopio, Finland; 3 Health Services & Policy Research, Exeter Collaboration for Academic Primary Care (APEx), NIHR PenCLAHRC, University of Exeter, Exeter, UK; 4 Department of General Practice and Elderly Care Medicine, Amsterdam UMC, Amsterdam, The Netherlands

**Keywords:** Multimorbidity, Person-centred care, Needs, Health service utilisation, Quality of life

## Abstract

**Background:**

A proactive person-centred care process is advocated for people with multimorbidity. To that aim, general practitioners may benefit from support in the identification of high-need patients, i.e. patients who are high or suboptimal users of health services and/or have a poor quality of life. To develop such support, we examined whether knowledge about patients’ illness perceptions and personal resources to manage their health and care is useful to identify high-need patients among multimorbid general practice populations.

**Methods:**

Survey data, collected in 2016 and 2017, of 601 patients with two or more chronic diseases (e.g. COPD, diabetes, Parkinson’s disease) registered with 40 general practices in the Netherlands were analysed by logistic regression analysis to predict frequent contact with the general practice, contact with general practice out-of-office services, unplanned hospitalisations and poor health related quality of life. Patients’ illness perceptions and personal resources (education, health literacy, mastery, mental health status, financial resources, social support) were included as predictors.

**Results:**

The four outcomes were only weakly associated among themselves (Phi .07–.19). Patients’ illness perceptions and personal resources were of limited value to predict potentially suboptimal health service use, but they were important predictors of health related quality of life. Patients with a poor health related quality of life could be identified by their previously reported illness perceptions (attributing many symptoms to their chronic conditions (B = 1.479, *P* < .001), a high level of concern (B = 0.844, *P* = .002) and little perceived control over their illness (B = -0.728, *P* = .006)) combined with an experienced lack of social support (B = -0.527, *P* = .042) and a poor mental health status (B = -0.966, *P* = .001) (sensitivity 80.7%; specificity 68.1%).

**Conclusions:**

Multimorbid patients who frequently contact the general practice, use general practice out-of-office services, have unplanned hospitalisations or a poor health related quality of life are largely distinct high-need subgroups. Multimorbid patients at risk of developing a poor quality of life can be identified from specific illness beliefs, a poor mental health status and unmet social needs.

## Background

Multimorbidity, defined as the co-existence of two or more chronic conditions in a person [1], has climbed high on the health policy agenda in many countries, as there is increasing awareness of the magnitude it has on individuals and society [2]. Multimorbidity puts a high burden on the life of patients and their families and impacts their functioning, mental health, social relationships, labour participation and financial situation [3–6]. Together with the rapid increase of the multimorbid population [7, 8], this also affects countries’ workforce and welfare [9]. In addition, healthcare expenditures related to multimorbidity are high. A recent study in the United Kingdom found multimorbid general practice patients (27% of the adult general practice population) to account for 53% of all consultations with general practitioners (GPs), 56% of the hospital admissions and 79% of all prescribed medicines [10]. Other studies show similar findings (e.g., [11–13]).

Over the last decade the quality of care for multimorbid patients has become a major concern, as care delivery is often fragmented and uncoordinated, resulting in duplication, inefficiency and suboptimal outcomes [14, 15]. Awareness has raised that managing multimorbidity asks for a person-centred approach [15, 16], as the outcomes to strive for are not straightforward and priorities should be set together with the patient based on his/her personal values and circumstances. This requires care professionals to tailor their care to individual needs [17]. In countries where patients are registered with a GP, it is in particular the GP who could play a key role in providing person-centred care. For instance, by developing an individual care plan with the patient, coordinating care or helping the patient to connect with social care and community services. To initiate a person-centred care process proactively, GPs might benefit from support to identify high-need patients among their multimorbid patient population, because of their risk of adverse outcomes such as uncontrolled health problems resulting in a high or suboptimal use of health services (e.g., frequent visits to the general practice, use of general practice out-of-office services, emergency department visits, unplanned hospitalisations) or a poor quality of life.

Inspired by a study in which older patients at risk of developing a frail health status were identified in Dutch general practice [18], we developed an algorithm to search general practice electronic health records (EHRs) to identify multimorbid patients with a high risk of potentially suboptimal use of health services [19]. As information registered in patients’ EHRs could not predict future unplanned hospitalisation and we also wanted to identify patients with a high risk of developing a poor quality of life, we conducted a second study to find patient reported predictors of high needs among multimorbid patients, for the purpose of developing a short screening questionnaire that could (additionally) be used in daily general practice. Potential (patient reported) indicators we studied were patients’ illness perceptions and personal resources to manage their health and care.

*Illness perceptions* are patients’ personal beliefs about the cause(s) of their illness, the identity of their condition(s) and symptoms attributed, the impact of their illness on their life, whether and how the course of illness could be influenced by medical treatment or their own behaviour, emotional representations and concern [20]. Illness perceptions have proven to be associated with patients’ health behaviours [21–23], although the prospective value of single illness perception dimensions for patients’ self-management has not been demonstrated [24]. A recent study showed an external local of control, i.e. a belief that life events are outside one’s control, to be an important predictor of developing multimorbidity over 10 years [25]. Illness perceptions of chronic patients have also been found to relate to patients’ quality of life (e.g. [26, 27]), clinical outcomes (e.g. [28]) and use of health services (e.g. [29, 30]). Among people with multimorbidity interacting control beliefs and perceived consequences appeared to predict their physical functioning over 6 months [31].

Patients’ ability to manage their health and care may depend on the *resources* they have at their disposal, such as education, income, social support and health literacy. Previous studies among populations with chronic conditions have demonstrated that a low education level and low income relate to worse clinical outcomes and a poor quality of life [32–34]. Specifically among multimorbid populations it was found that people who live alone and/or have a low level of health literacy are more likely to experience a poor quality of life [35]. .This study had a cross-sectional design, which did not allow to establish the predictive value of persons’ resources. Moreover, it did not study relations between personal resources and use of health services.

### Research questions

1. To what extent are specific illness perceptions and personal resources of multimorbid patients independent predictors of potentially suboptimal use of health services and/or a poor health related quality of life?
2. Which combinations of illness perceptions and personal resources best predict these outcomes among multimorbid general practice patients, in addition to their demographic and medical characteristics?

## Methods

### Study design and sample

We conducted a longitudinal study, analysing survey data from multimorbid patients who participated in a nationwide panel-study in the Netherlands [5, 36]. Patients with chronic disease(s) are recruited each year from general practices throughout the Netherlands to participate in the panel-study, based on the following criteria: diagnosis of somatic chronic disease(s), aged 15 years or older, not institutionalised, life expectancy longer than 6 months according to the GP, mentally able to participate and adequate command of the Dutch language. Participants complete questionnaires every 6 months for a maximum of 4 years. GPs provide data about the chronic disease(s) registered as ICPC-1 codes [37] with permission of the participants. The panel-study is registered with the Dutch Data Protection Authority (registration no. 1283171); all data are collected and handled in accordance with the privacy protection guidelines of the Authority.

In this study we included participants who responded to three successive surveys (April and October 2016, April 2017), of which the most recent one included the outcome measures of health service utilisation and health related quality of life, and the previous surveys included measures of patients’ illness perceptions (April 2016) and resources (April 2016, except of available social support and health literacy, of which measures were included in the survey of October 2016). Furthermore, participants had to be diagnosed with at least two chronic diseases according to the list developed by O ‘Halloran and colleagues [38]. As the panel-study does not require GPs to provide data about participants’ mental health problems (P-chapter of the ICPC), chronic psychiatric or mental health disorders were not taken into account.

### Data collection

#### Outcome measures

*Potentially suboptimal use of health services*: In April 2017 participants were asked to report: 1. their number of contacts with the GP or the practice nurse in 2016 (including home visits and consultations by telephone; excluding calls for repeat prescriptions), 2. whether they had contact with general practice out-of-office services by telephone or face-to-face in 2016, 3. whether they had one or more unplanned hospitalisations in 2016. For general practice consultations, we chose the cut-off point of ≥ nine contacts with the GP or practice nurse based on the distribution in the sample, as we could not rely on (inter-)national consensus or other studies to designate a certain number of contacts as potentially suboptimal. Frequent contact with the general practice may point to unmet needs, perhaps also in the psychological or social domain, whereas acute care use may be preventable to some extent by early detection of health risks and adequate follow-up care. However, we wish to emphasise that frequent contact with the general practice, using general practice out-of-office services and unplanned hospitalisations may be suboptimal from the perspective of proactive care, but do not automatically point to inappropriate use of services, as this will depend on the necessity and urgency to receive medical care in a specific situation. Therefore we use the term ‘potentially suboptimal’ in this article.

*Poor health related quality of life* was assessed by the EQ-5D-5L [39], which consists of five items assessing mobility, self-care, performance of usual activities, pain/discomfort, and anxiety/depression, each with five response options. Based on the preferences of a representative Dutch general population sample, index values were assigned to each of the 3125 (5^5^) possible health states, ranging from − 0.446 to 1 [40]. As a formal cut-off point does not exist, we operationalised a poor health related quality of life as a score lower than .67 (boundary of percentile-20 in our study), which is one standard deviation below the reference value of the Dutch general population aged 50 and older [40].

#### Predictors

Patients’ *illness perceptions* were assessed by the Brief Illness Perception Questionnaire (BIPQ) [41]. This questionnaire consists of eight items, each referring to another dimension of a person’s illness representation, based on the Common Sense Model of self-regulation [20]: personal beliefs about the consequences of one’s illness, about the duration, the perceived ability to control the illness by one’s own behaviour, beliefs about the extent to which medical treatment helps to control the illness, symptoms experienced and attributed to the disease (identity), concerns, perceived understanding of one’s illness, and one’s emotional response to the illness. In the survey, multimorbid patients were asked to respond to the questions keeping in mind the condition they were suffering from most.

*Personal resources* were assessed by a set of survey questions measuring various resources:

- Social resources: Participants reported whether they lived together with a spouse or partner. In addition, we included one item of the Loneliness scale [42]: “There are plenty of people I can lean on when I have problems”, with three response options (yes, more or less, no).
- Education: Level of education was based on the highest level of completed education. We distinguished three categories: low (primary school or low/preparatory vocational training), medium (intermediate or advanced general education or intermediate vocational training), and high (high vocational education or university).
- Financial resources: We included one item about one’s financial situation: “How would you describe your current financial situation?” (answering options: 1: I have to make debts, 2: I need to use my savings, 3: I get by, 4: I save some money, and 5: I save a lot of money) [43].
- Health literacy: Health literacy has been described by the WHO as “the cognitive and social skills which determine the motivation and ability of individuals to gain access to, understand and use information in ways which promote and maintain good health” [44]. We included the Health Literacy Questionnaire (HLQ) [45], which consists of 44 items belonging to nine scales, based on a comprehensive model of health literacy (see Table 2). Five scales have a four-response format (strongly disagree, disagree, agree, strongly agree) and four a five-response format (cannot do, very difficult, quite difficult, quite easy, very easy). Scale scores could range between 1 and 4 for the scales with four response options, and between 1 and 5 for those with five response options. Higher scale scores refer to higher levels of health literacy. Cronbach’s alpha coefficients of the nine scales in our study ranged from .71 to .90, indicating good internal consistency.
- Mastery: Mastery is a psychological resource and has been defined as “the extent to which one regards one’s life-chances as being under one’s own control in contrast to being fatalistically ruled” [46]. We included a Dutch version [47] of the Pearlin Mastery Scale [46], which consists of five negatively worded and two positively worded items. Unlike the original scale, the Dutch items are scored on a five-response format, from 1 (strongly disagree) to 5 (strongly agree). Scale scores could range between 7 and 35, with higher scores indicating higher levels of mastery. Cronbach’s alpha in our study was .81.
- Mental health: To assess mental health, we included the Depression scale of the Hospital Anxiety and Depression Scale (HADS) [48]. This scale consists of seven items with four answering options, ranging from 0 to 3. Scale scores could range from 0 to 21; a scale score of 8 or higher is considered an indication of a mild or more severe depression [49]. Cronbach’s alpha of the scale in our study was .83.

### Statistical analyses

We conducted univariate analyses to describe the demographic and medical characteristics of the participants as well as their use of health services and health related quality of life. Subsequently, we determined cut-off scores on the measures of patients’ illness perceptions and personal resources (see Supplementary file 1). Next, we assessed the associations (Phi coefficients) between the four outcome variables. As these associations appeared to be weak (Phi ranging from .07 to .19), we analysed each outcome variable separately.

To answer our first question, we conducted logistic regression analysis for each outcome variable as the dependent variable and each illness perception dimension or resource separately included as predictor. To answer our second question, we first checked for collinearity (see Supplementary file 1). Next, we conducted two preparatory multivariate logistic regression analyses (see Supplementary file 1) to limit the number of predictors compared to the number of cases in the final multivariate model. In the final multivariate logistic regression model we conducted for each outcome variable we included the patient’s age, sex, number of chronic diseases and those illness perception dimensions and resources that had proven to be significant predictors in the bivariate or preparatory multivariate analyses. ROC curves were constructed to assess the specificity and sensitivity of the final prediction models and the area under the ROC curve (AUC). In case of AUC greater than .70, we determined the most appropriate cut-off value based on the coordinates of the curve, considering that we aimed to predict at least 80% of the true positives, while also maximizing the proportion of true negatives.

## Results

### Participants and drop-out

**Fig. 1:**
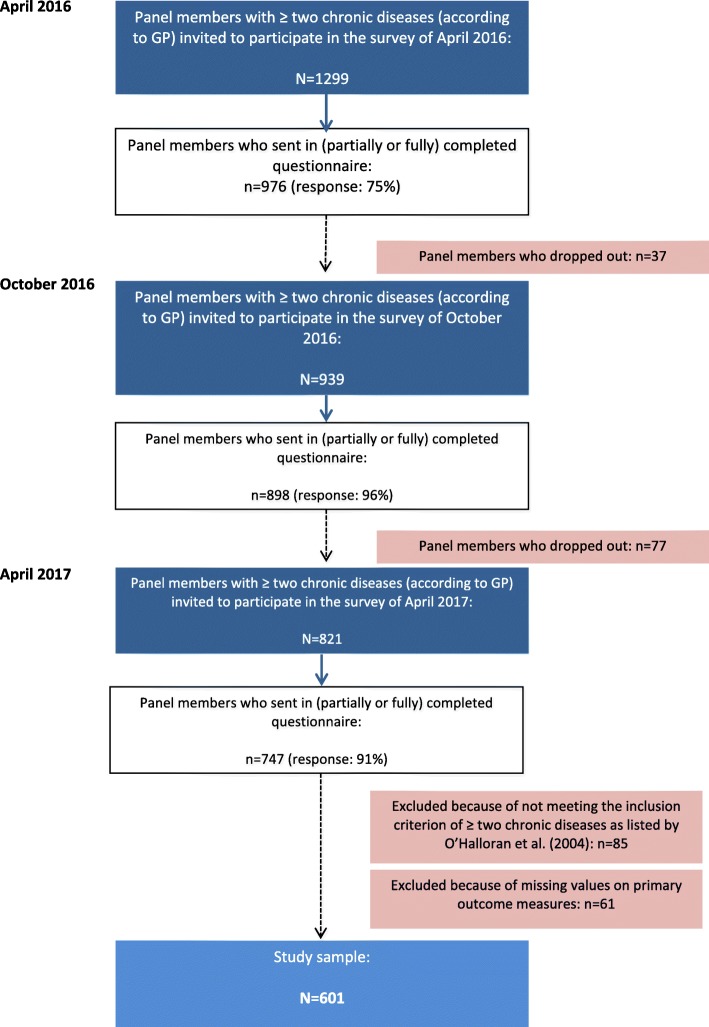
Flowchart visualising the sample selection steps

Of the 1299 panel members with two or more chronic diseases, 747 participated in all three surveys (response rates: April 2016 75%, October 2016 96%, April 2017 91%) (see Fig. 1). Apart from once-only non-response, 114 persons (9%) dropped out the panel-study in-between because of various reasons: they felt the questions did not apply to them (*n* = 25), were too difficult (*n* = 18) or too intimate (*n* = 6), they felt too ill (*n* = 15), they had reached the participation term of 4 years (*n* = 11), because of death (*n* = 4), not interested anymore (n = 6), personal reasons (n = 4) or unknown (n = 25). In the latter case it concerned persons who did not respond to two successive surveys. Of the 747 responders to all three surveys, we excluded 85 persons because they did not have at least two chronic diseases of the list developed by O’Halloran and colleagues, and 61 because of missing values on the outcome measures. This resulted in a final sample of 601 persons, from 40 general practices throughout the Netherlands.

### Sample characteristics, use of health services and health related quality of life

Gender distribution was 52% women and 48% men. The mean age was 69.2 years (SD 9.9 years). Half of the sample (51%) had been diagnosed with two chronic diseases, 30% with three, and 19% with four or more chronic diseases. Hypertension (34%), diabetes (29%) and ischemic heart disease (21%) had been diagnosed most frequently, followed by osteoarthritis (14%), asthma (14%), atrial fibrillation (12%), COPD (11%) and hypothyroidism (11%).

**Fig. 2:**
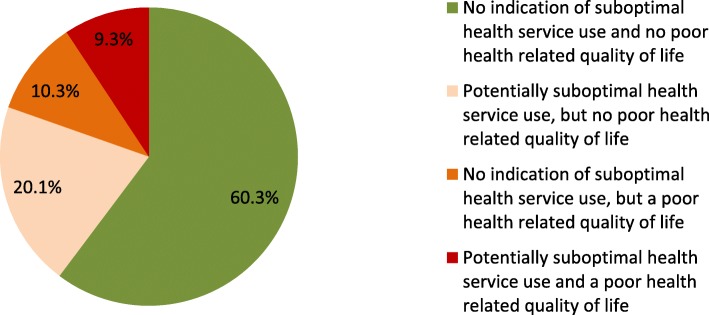
Distribution of multimorbid patients according to health service use and health related quality of life (*N* = 601)

The average number of contacts with the general practice in 2016 was 6.6; 23% reported nine or more contacts (Table 1). 21% reported to have had contact with the general practice out-of-office service; 9% reported an unplanned hospital admission in 2016. The mean EQ-5D-5 L score was 0.77, with 20% of the sample scoring below 0.67 (‘poor health related quality of life’). 60% of the sample did not have an indication of potentially suboptimal use of health services or a poor health related quality of life (Fig. 2). Of the remaining 40%, half had one or more indications of potentially suboptimal use of health services but not a poor health related quality of life.

**Table 1:** Use of health services and health related quality of life of participating multim006Frbid patients

	N	Mean (SD)	n	%
**Use of services in previous year***(self-reported; April 2017)*				
Number of consultations general practice (GP and practice nurse):	593	6.56 (6.45)		
0			25	4.2
1 to 4			220	37.1
5 to 8			213	35.9
9 to 12			84	14.2
More than 12			51	8.6
Contact with general practice out-of-office service: (ref. no contact)	601		124	20.6
only by telephone			35	5.8
(also) consultation(s)/visit(s)			89	14.8
Hospital admission(s): (ref. no)	597			
Yes			104	17.4
Unplanned hospitalisation(s): (ref. no)	596			
Yes			54	9.1
Total number of nights in hospital	588			
0			493	83.8
1 to 4			60	10.2
5 or more			36	6.1
**Health related quality of life***(EQ-5D index value; April 2017)*	601	0.77 (0.22)		
Poor health related quality of life (index value < 0.67)	601		118	19.6

### Illness perceptions and resources

Participants’ illness perception scores showed high levels of understanding of their main condition and relatively low levels of concern and emotional response. Personal control beliefs were rather low (Table 2), indicating that most participants did not believe to have much influence on the course of their illness(es). Regarding their personal resources, about three quarters lived together with a partner. Most participants reported sufficient financial means, but 15% made debts or had to use savings to make ends meet. With regard to health literacy, most difficulties related to finding good health information, critically appraising the information and resources, and finding out about available services and support (‘navigating the healthcare system’). Almost one fifth had an indication of a depression.

**Table 2:** Illness perceptions and personal resources of participating multimorbid patients

Illness perceptions	N	Mean (SD)	N	%
Consequences (0–10)	584	5.39 (2.62)		
Consequences dichotomous (score ≥ 7)			245	42.0
Timeline (0–10)	582	9.03 (1.85)		
Timeline dichotomous (score ≥ 7)			539	92.6
Personal control (0–10)	584	5.92 (2.41)		
Personal control dichotomous (score ≥ 7)			288	49.3
Treatment control^a^ (0–10)	460	6.72 (2.18)		
Treatment control dichotomous (score ≥ 7)			290	63.0
Identity (0–10)	585	5.38 (2.64)		
Identity dichotomous (score ≥ 7)			250	42.7
Concern (0–10)	571	4.79 (2.78)		
Concern dichotomous (score ≥ 7)			185	32.4
Understanding (0–10)	575	7.52 (2.33)		
Understanding dichotomous (score ≥ 7)			436	75.8
Emotional response (0–10)	577	4.12 (2.91)		
Emotional response dichotomous (score ≥ 7)			156	27.0
**Personal resources**				
***Social resources***				
Lives with partner	597		441	73.9
There are plenty of people I can lean on when I have troubles	580			
No			38	6.6
More or less			144	24.8
Yes			398	68.6
***Education level***	584			
Low (primary school or low/preparatory vocational training)			205	35.1
Medium (intermediate or advanced general education or intermediate vocational training)			246	42.1
High (applied university, university)			133	22.8
***Financial resources***				
How would you describe your current situation?	570			
I have to make debts			13	2.3
I need to use my/our savings			72	12.6
I get by			191	33.5
I save some money			247	43.2
I save a lot of money			47	8.2
***Health literacy***				
Healthcare provider support (HPS) (1–4)	583	3.04 (0.41)		
HPS dichotomous (score ≥ 3)			450	77.2
Having sufficient information (HSI) (1–4)	576	2.95 (0.34)		
HSI dichotomous (score ≥ 3)			419	72.7
Actively managing health (AMH) (1–4)	570	2.81 (0.39)		
AMH dichotomous (score ≥ 3)			275	48.2
Social support (SS) (1–4)	577	2.92 (0.43)		
SS dichotomous (score ≥ 3)			360	62.4
Critical appraisal (CA) (1–4)	570	2.60 (0.47)		
CA dichotomous (score ≥ 3)			182	31.9
Active engagement with healthcare providers (AE) (1–5)	559	3.88 (0.64)		
AE dichotomous (score ≥ 4)			328	58.7
Navigating the healthcare system (NHS) (1–5)	563	3.74 (0.65)		
NHS dichotomous (score ≥ 4)			244	43.3
Ability to find good health information (FHI) (1–5)	557	3.72 (0.67)		
FHI dichotomous (score ≥ 4)			259	46.5
Reading and understanding health information (UHI) (1–5)	560	3.83 (0.61)		
UHI dichotomous (score ≥ 4)			287	51.3
**Mastery**	581			
Mastery scale score (7–35)		23.40 (5.11)		
Mastery scale score > 21			379	65.2
**Mental health**	584			
HADS depression scale score (0–21)		4.48 (3.76)		
High-risk major depression (scale score ≥ 8)			112	19.2

^a^ only filled in if applicable (perceived by participants as in case of medical treatment); *n* = 94 chose the n.a.-option

### Results of bivariate analyses

None of the illness perception dimensions was a significant predictor of more than one indicator of potentially suboptimal use of health services (Table 3), except participants’ emotional response to their illness, which increased both the likelihood of frequent contact with the general practice and of contact with general practice out-of-office services. Control beliefs were the only significant predictor of unplanned hospitalisations: people who perceived high control over their illness, were less likely to report an unplanned hospitalisation. In contrast, all illness perception dimensions, except perceptions regarding the timeline, were significant predictors of a poor health related quality of life. Similarly, many resource variables were significant predictors of a poor health related quality of life, whereas none of these significantly predicted the use of general practice out-of-office services and unplanned hospitalisations. Having limited personal resources appeared to predict frequent contact with the general practice, in particular having a low level of health literacy (experiencing difficulties in navigating the healthcare system and in reading and understanding health information), lacking a sense of mastery and feeling depressed.

**Table 3:** Illness perception dimensions and resources as predictors of potentially suboptimal use of services and/or a poor health related quality of life, results of multinomial logistic regression analyses with one predictor included per analysis

	**Frequent contact with GP**		**Use of general practice out-of-office service**		**Unplanned hospitalisation**		**Poor health related quality of life**	
	**N**	**OR**	**N**	**OR**	**N**	**OR**	**N**	**OR**
***Illness perceptions***								
BIPQ Consequences ≥7 (vs < 7)	576	1.21	584	1.42	580	1.56	584	5.68***
BIPQ Timeline ≥7 (vs < 7)	574	1.81	582	0.87	578	0.98	582	2.56
BIPQ Personal control ≥7 (vs < 7)	576	0.90	584	1.01	580	0.40**	584	0.37***
BIPQ Treatment control ≥7 (vs < 7)	455^a^	0.66	460^a^	0.64*	457^a^	0.61	460^a^	0.29***
BIPQ Identity ≥7 (vs < 7)	577	1.73**	585	1.13	581	1.27	585	7.10***
BIPQ Concern ≥7 (vs < 7)	564	1.94**	571	1.18	566	1.46	571	6.58***
BIPQ Understanding ≥7 (vs < 7)	568	1.16	575	0.84	570	0.70	575	0.85
BIPQ Emotional response ≥7 (vs < 7)	570	2.02**	577	1.70*	572	1.72	577	5.55***
***Personal resources***								
Education level: medium (vs low)	576	0.91	584	0.86	580	0.82	584	0.79
Education level: high (vs low)	576	0.79	584	0.95	580	0.74	584	0.38**
“I get by or I save” (vs “I have to make debts or use my savings”)	562	0.66	570	1.20	566	1.26	570	0.42**
Lives with partner	589	1.00	597	1.24	592	1.25	597	0.65*
“Have plenty people”: yes (vs more or less / no)	574	0.75	580	1.09	575	0.94	580	0.41***
HLQ Healthcare provider support: score ≥ 3 (vs < 3)	577	1.05	583	0.62*	579	0.80	583	0.70
HLQ Having sufficient information ≥3 (vs < 3)	569	0.70	576	0.75	571	0.81	576	0.51**
HLQ Actively managing health ≥3 (vs < 3)	563	1.16	570	0.77	565	1.28	570	1.17
HLQ Social support ≥3 (vs < 3)	569	1.02	577	1.32	572	1.02	577	0.96
HLQ Critical appraisal ≥3 (vs < 3)	564	1.21	570	0.67	566	0.87	570	0.90
HLQ Active engagement with healthcare providers ≥4 (vs < 4)	553	0.87	559	0.68	555	1.24	559	0.43***
HLQ Navigating the healthcare system ≥4 (vs < 4)	557	0.62*	563	0.75	559	0.82	563	0.48**
HLQ Ability to find good health information ≥4 (vs < 4)	551	0.73	557	0.72	553	1.07	557	0.54**
HLQ Reading and understanding health information: score ≥ 4 (vs < 4)	554	0.66*	560	0.77	556	0.76	560	0.58*
Mastery > 21 (vs ≤21)	574	0.51**	581	0.77	577	0.62	581	.20***
HADS-Depression < 8 (vs ≥8)	577	0.68	584	0.77	579	1.39	584	.16***

*HLQ* Health Literacy Questionnaire, *HADS* Hospital Anxiety and Depression Scale, *BIPQ* Brief Ilness Perception Questionnaire, *OR* Odds Ratio; * *p* < 0.05; ** *p* < 0.01; *** *p* < 0.001

^a^ only filled in if applicable (perceived by participants as in case of medical treatment); *n* = 94 chose the n.a.-option

### Results of multivariate analyses

Including all significant predictors from the previous analyses in one multivariate model (Table 4) showed that sex (female), a high level of concern and lacking a sense of mastery increased the likelihood of frequent contact with the general practice. The use of general practice out-of-office services was predicted by age (75+) and a strong emotional response to one’s condition(s). Perceiving high personal control over one’s illness decreased the likelihood of unplanned hospitalisations. Perceiving high control also decreased the chance of a poor health related quality of life, whereas attributing many symptoms to one’s condition(s) and a high level of concern increased the likelihood of experiencing a poor health related quality of life 12 months later. A belief that social support was sufficiently available as well as a good mental health status decreased the likelihood of a poor health related quality of life 12 months later.

**Table 4:** Final prediction models of potentially suboptimal use of health services and a poor health related quality of life, results of multivariate multinomial logistic regression analyses

	**Frequent contact with the general practice**	**Use of general practice out-of-office service**	**Unplanned hospitali-Sation**	**Poor health related quality of life**
**Final model**	Chi^2^(3) = 21.512 (*P* < .001)	Chi^2^(3) = 16.037 (*P* = .001)	Chi^2^(1) = 9.654 (*P* = .002)	Chi^2^(7) = 137.288 (*P* < .001)
N	552	577	580	541
	**B (*P*)**	**B (*P*)**	**B (*P*)**	**B (*P*)**
Sex: male (ref: female)	−0.493 (.019)			
Age (ref: younger than 65 years)				
65 to 74 years		0.187 (.490)		−0.477 (.131)
75 years or older		0.775 (.003)		0.458 (.135)
Number of chronic conditions				
BIPQ Personal control ≥7			−0.926 (.003)	−0.728 (.006)
BIPQ Identity ≥7				1.479 (<.001)
BIPQ Concern ≥7	0.446 (.048)			0.844 (.002)
BIPQ Emotional response ≥7		0.622 (.005)		
“I have plenty people”: yes (vs more or less/no)				−0.527 (.042)
HADS-Depression < 8				−0.966 (.001)
Mastery > 21	−0.533 (.017)			
Constant	−0.828 (<.001)	−1.838 (<.001)	− 1.904 (<.001)	− 1.279 (.002)
Hosmer-Lemeshow goodness of fit	Chi^2^(6) = 2.726 (*P* = .842)	Chi^2^(4) = 0.249, (*P* = .993)	Chi^2^(0) = 0.000 (−)	Chi^2^(8) = 3.401 (*P* = 0.901)

### Classification

Prediction of frequent contact with the general practice, use of general practice out-of-office services and unplanned hospitalisations was poor (AUC < .70), whereas prediction of poor health related quality of life by the final model was acceptable (AUC .837) (Table 5). Based on the coordinates of the curve provided by the ROC analysis, we determined a cut-off value of .16 most optimal, considering that we aimed to identify at least four out of five persons with a poor health related quality of life 12 months later, while also classifying correctly as many persons possible who did not experience a poor health related quality of life.

**Table 5:** Proportions of cases correctly predicted (sensitivity and specificity) for various classification cut-offs

	**Frequent contact with general practice**		**Use of general practice out-of-office service**		**Unplanned hospitalisation**		**Poor health related quality of life**	
**Final model**	Chi^2^(3) = 21.512 (*P* < .001)		Chi^2^(3) = 16.037 (*P* = .001)		Chi^2^(1) = 9.654 (*P* = .002)		Chi^2^(7) = 137.288 (*P* < .001)	
N	552		577		580		541	
AUC	.626		.612		.609		.837	
**Classification cut-off:**							**Sens.**^**a**^	**Spec.**^**b**^
.10							90.8	57.2
.15							81.7	67.6
.16							80.7	68.1
.17							79.8	69.7
.20							74.3	75.2
.25							67.9	82.2
.50							43.1	94.0

^a^ Sens. = Sensitivity: number of cases predicted with adverse outcome / total number of cases observed with adverse outcome

^b^ Spec. = Specificity: number of cases not predicted with adverse outcome / total number of cases not observed with adverse outcome

## Discussion

Multimorbid patients’ perceptions of their illness and personal resources appear of little value to predict who will frequently contact the general practice, use general practice out-of-office services or have unplanned hospitalisations. Assessing patients’ illness perceptions and resources may however be helpful to identify which multimorbid patients have a high risk of developing a poor health related quality of life. This applies in particular to certain illness beliefs (attributing many symptoms to one’s chronic condition(s), being very concerned and experiencing little control over one’s illness) and resources (a poor mental health status, little social support).

The predictive value of patients’ illness perceptions for quality of life is in line with previous cross-sectional and longitudinal studies among chronic patients [26, 27, 50, 51]. This also holds for the predictive value of specific illness perception dimensions for health service use [52]. For instance, we found patients’ level of concern to be a significant predictor of frequent contact with the general practice, which is in line with a study among survivors of endometrial cancer, which showed survivors with higher levels of concern to be more likely to visit their GP or medical specialist [53]. Considering the role of patients’ resources, we found many to be significant predictors of health related quality of life in the bivariate analyses, which is in line with other studies (e.g. [32–35, 54]), but few remained their significance in the multivariate model. Moreover, hardly any resources we included were significant predictors of potentially suboptimal service use. The bivariate models show several aspects of health literacy and mastery to be predictive of (not) frequently contacting the general practice or using out-of-office services, which is in line with other studies [35, 55], but only mastery remained its significance in the multivariate model predicting frequent contact with the general practice.

An important finding of our study is that multimorbid patients at risk of a poor health related quality of life are for a large part not the same patients who frequently contact the general practice, visit the general practice out-of-office service or have unplanned hospitalisations, or vice versa. This also explains why hardly any illness perception dimensions or resources were of predictive value for more than one of these outcomes.

The poor prediction of frequent contact with the general practice, contact with general practice out-of-office services and unplanned hospitalisations may be explained by either the choice of predictors or the nature of these outcomes. To start with the first option, frequent contact with the general practice may be better predicted by data registered in patients’ general practice EHR, alone or in combination with patient-reported information, as in healthcare systems where GPs play a big role in chronic disease management contact with the general practice will for a large part not be initiated by the patient. As we mentioned in the introduction, we aimed to develop support for GPs to identify multimorbid patients with a high need for proactive person-centred care based on a two-step procedure: first, applying an algorithm that automatically searches patients’ EHRs for indicators of potentially suboptimal use of health services; then applying a short screening questionnaire to assess the illness perceptions and personal resources of the patients selected by the first step, to improve identification of high needs, also taking into account patients’ quality of life. Our study to develop the algorithm for the first step [19] confirms that data registered in patients’ general practice EHRs are good predictors of future frequent contact with the general practice, but not of unplanned hospitalisations. This brings us to the second option, which may apply in particular to the use of general practice out-of-office services and unplanned hospitalisations: the future use of these services by multimorbid patients may be difficult to predict anyway, regardless of which patient data, clinical or self-reported, we use. Due to the complexity of multimorbidity and related medical treatment (e.g. polypharmacy), unexpected health problems may arise, which necessitates acute care.

### Strengths and limitations

Participants were selected from a nationwide panel-study, which uses a standardised procedure to randomly select general practices throughout the country, and within these practices, patients with chronic diseases [5]. Although we initially selected 1299 panel members with two or more chronic conditions registered by GPs, our final sample was substantially smaller (N = 601), as for this longitudinal study we only included persons who had responded to three successive surveys over a year, with two or more chronic diseases defined as such by O’Halloran and collleagues (2004) and who did not have missing data on any of the four outcome measures. Statistical analyses comparing the background characteristics of our final sample with the group of excluded persons demonstrated that the two groups had similar distributions of gender, education level and household composition (*P* > .05). However, our final sample consisted of more people with three or more chronic diseases (49% vs 40%) and less people with two chronic diseases (51% vs 60%) than the group of persons not included in the final sample. Moreover, our final sample was on average slightly older (69,2 years) than the group op persons not included in the final sample (67,3 years). These differences could be explained by respondents who appeared not to have two or more chronic diseases that were recognised as such by O’Halloran et al. (2004), whom we purposefully excluded from the sample before starting data-analysis. Given that the latter respondents did not belong to the study population we aimed to include, we believe our sample to be a good representation of the population we envisaged for this study.

The comprehensive half-yearly surveys provide rich data about participants’ illness perceptions and resources, which is very valuable in combination with registration data of chronic diseases.

As there is no valid questionnaire available in Dutch, we used the BIPQ, which has been designed to assess the patient’s perception of a single disease or condition. Our multimorbid participants were asked to complete the BIPQ keeping in mind the condition they were suffering from most. This may have resulted in an incomplete picture of their perception of the multiple diseases they have, and possibly also a lack of insight in aspects that are particularly relevant for their perception of multimorbidity, for instance beliefs about common causal factors or interactions between conditions or treatments. On the other hand, many people with multimorbidity seem unable to disentangle their conditions [56], as their perception of illness is different from healthcare professionals’ disease constructs [57].

Furthermore, the self-reported use of health services may be considered a limitation of the study. Participants were asked in April 2017 to report the number of contacts with the general practice, the use of general practice out-of-office services and any unplanned hospitalisations in 2016. Recall bias may have occurred. Comparing our self-reported data with data of more than 200,000 multimorbid patients registered in a general practice database and hospital registrations in 2013 show nevertheless high resemblance: in this study we found 23% with nine or more contacts with the general practice and 9% with unplanned hospitalisations, whereas this was respectively 20 and 7% in the registered data of 2013 [19].

Another issue is that participants reported in April 2017 their use of health services in 2016, while data about their illness perceptions and personal resources were collected in 2016. Given that data about illness perceptions and resources were predominantly collected by a survey in April 2016, it seems likely that the largest part of their health service use in 2016 took place after this survey. It is possible however that participants had contact with the general practice out-of-office service or had an unplanned hospitalisation before assessment of their illness perceptions and resources. Illness perceptions and resources are usually however rather stable, once developed.

A final remark concerns the need to validate our results. Given its size, we could not randomly split the sample to create two data sets, to respectively build and validate our prediction models. We recently initiated new data collection among adult patients with multimorbidity who participate in a quality improvement initiative of a group of general practices in the Netherlands for the purpose of validation.

### Recommendations for future research

Some of the limitations of our study may be solved by future research. First, there is a need to develop and validate a questionnaire for people with multimorbidity that covers the broad spectrum of their perceptions of multimorbidity. Promising work has been done in the United Kingdom with the development of the Multimorbidity Illness Perceptions Scale (MULTIPleS) [58], but further testing of its construct and predictive validity is needed. Moreover, translation and cross-cultural validation will be necessary in order to make this questionnaire suitable for use in other countries. Second, to identify high-need patients it would be a big step forward to link clinical registration data and patient-reported data of general practice patients with multimorbidity and analyse these data together. In this way it could be more reliably assessed whether people with high needs as reflected by their use of particular health services are indeed different from people with high needs as reflected by patient-reported outcomes, such as health related quality of life. Finally, we wish to emphasise the necessity to conduct effectiveness studies on integrated care pathways among high-need patient populations. There is still limited evidence of which types of (integrated) care are effective to improve health related outcomes and optimal use of health services among multimorbid patient populations [59, 60]. Such evidence is of utmost importance, as identifying high-need patients only makes sense, if there is evidence based support for GPs on which care these people are most likely to benefit from.

### Relevance for clinical practice

Based on this study, we designed a brief screener to identify persons who are likely to develop a poor health related quality of life in the near future among adults with two or more chronic conditions, which could be applied by the GP or practice nurse together with the patient. This screener consists of seven questions: four derived from the BIPQ (perceived consequences, personal control, concern and emotional response) [41], one from the HADS-Depression scale [48] and two self-developed items on mastery and perceived social support. Item scoring and the classification cut-off score are based on the current study. The screening result could be used to invite patients for one or more person-centred consultations and/or follow-up care, as agreed upon with the patient. Feasibility of using the screener is currently being piloted.

Irrespective of using a screener, we strongly advise healthcare professionals to explore the illness perceptions and personal resources of their patients with multimorbidity regularly, in particular their control beliefs, attributed symptoms, concerns and emotional response to their illness and their support network, mental health and sense of mastery. Financial issues related to service use and health literacy may also be explored in subgroups of patients or deprived areas.

## Conclusions

People with multimorbidity at risk of a poor health related quality of life are for a large part not the same people who frequently contact the general practice, visit the general practice out-of-office service or have unplanned hospitalisations, or vice versa. As these four high-need subgroups show different characteristics, there is no common set of illness beliefs or personal resources that satisfactorily predict all four outcomes. Persons with multimorbidity who have a high risk of developing a poor health related quality of life can be identified by their specific illness beliefs (attribution of many symptoms to their chronic conditions, a high level of concern and little perceived control over their illness), a poor mental health status and unmet social needs.

## Supplementary information

**Additional file 1.** Detailed description of statistical analyses.

## Data Availability

The dataset analysed for the current study is available from the corresponding author on reasonable request.

## Abbreviations

AE (subscale): Active Engagement with healthcare providers (Health Literacy Questionnaire)
AMH (subscale): Actively Managing Health (Health Literacy Questionnaire)
AUC: Area Under the Curve
BIPQ: Brief Illness Perception Questionnaire
CCMO: Central Committee on Research Involving Human Subjects
COPD: Chronic Obnstructive Pulmonary Disease
CA (subscale): Critical Appraisal (Health Literacy Questionnaire)
EHR: Electronic Health Record
EQ-5D: EuroQuol 5 Dimensions questionnaire
EQ-5D-5 L: 5-level EQ-5D version
FHI (subscale): ability to Find good Health Information (Health Literacy Questionnaire)
HADS: Hospital Anxiety and Depression Scale
HPS (subscale): Healthcare Provider Support (Health Literacy Questionnaire)
HSI (subscale): Having Sufficient Information (Health Literacy Questionnaire)
HKQ: Health Literacy Questionnaire
ICPC: International Classification of Primary Care
GP: general practitioner
MULTIPleS: Multimorbidity Illness Perceptions Scale
N: number
NHS (subscale): Navigating the Healthcare System (Health Literacy Questionnaire)
NPCD: National Panel of people with Chronic illness or Disability
OR: Odds Ratio
ROC (analysis): Receiver Operating Characteristic (analysis)
SD: standard deviation
Sens.: Sensitivity
Spec.: Specificity
SS (subscale): Social Support (Health Literacy Questionnaire)
UHI (subscale): reading and Understanding Health Information (Health Literacy Questionnaire)
WHO: World Health Organization
WMO: Medical Research Involving Human Subjects Act
