# Supplementary material for: Identifying high-need patients with multimorbidity from their illness perceptions and personal resources to manage their health and care: a longitudinal study
Source: BMC Fam Pract. 2020 Apr 29;21:75. doi: 10.1186/s12875-020-01148-3 (PMC7191697; doi:10.1186/s12875-020-01148-3)
Supplement: Supplementary file 1 — Additional file 1. Detailed description of statistical analyses. [file 12875_2020_1148_MOESM1_ESM.docx]

**Supplementary file 1:** Detailed description of statistical analyses

1. Univariate analyses were conducted to describe the demographic and medical characteristics of participants as well as their use of health services and health related quality of life.
2. We determined cut-off scores of the potential predictor variables (illness perceptions and personal resources), as we aimed to develop a screener. Apart from the HADS depression score, no cut-off scores had been determined in other studies. Therefore, cut-off scores were based on common sense (e.g., combining ”agree” and ”strongly agree” versus ”disagree” and ”strongly disagree”), theoretical considerations (e.g., considering a score of 7 or higher as relevant for perceived personal control), practical considerations (e.g., applying the same cut-off score for all BIPQ items), and statistical analysis. Regarding the latter, we estimated the B coefficient and level of significance to predict frequent contact with the general practice, use of general practice out-of-office services, unplanned hospitalisations and a poor health related quality of life for each variable separately by means of logistic regression analysis, applying various scores as cut-off points (for instance, 6, 7 and 8 for the BIPQ items).
3. We assessed the associations (Phi coefficient) between the four binary outcome variables created in this way.
4. To answer our first research question, we conducted logistic regression analyses for each of the four outcome variables as the dependent variable and each illness perception dimension or resource variable included as predictor (analyses for each predictor separately).
5. To answer our second question, we first checked for collinearity by evaluating Spearman correlations between all illness perception dimension and resource variables and collinearity diagnostics (Tolerance and Variance Inflation Factor).
6. As we wanted to limit the number of predictor variables (compared to the number of cases) in the final multivariate analyses, we conducted two preparatory multivariate logistic regression analyses to predict a poor health related quality of life. In the first one, HLQ scale scores that appeared significant predictors in the bivariate analyses were entered using a forward stepwise procedure; HLQ scales that were selected by the stepwise procedure were included in the final model. A similar analysis was conducted with the BIPQ items (except Treatment control) that appeared significant predictors in the bivariate analyses. Treatment control was not entered, because many respondents (n=94) stated the item was not applicable to them, as they did not consider themselves to receive medical treatment. These preparatory analyses were not conducted for the three health service use variables, as only a small number of HLQ and BIPQ variables appeared to be significant predictors of these outcomes in the bivariate logistic regression analyses.
7. Finally, multivariate logistic regression analysis was conducted for each of the four outcome variables, entering age, sex, number of chronic conditions and all illness perception and resource variables that had proven to be significant predictors in the bivariate or preparatory multivariate analyses. A stepwise forward procedure (entry testing based on the significance of the score statistic, and removal testing based on the probability of the Wald statistic) was applied to construct the final prediction model for each of the four outcomes.
8. ROC curves plotting the predicted outcomes versus the observed outcomes were constructed for each of the outcomes to assess the specificity and sensitivity of the final models and the area under the ROC curve (AUC). In case of AUC greater than .70, we determined the most appropriate cut-off point based on the coordinates of the curve, considering that we aimed to predict at least 80% of the true positives, while also maximizing the proportion of true negatives.
